# Pattern of malignant solid tumors and lymphomas in children in the east delta of Egypt: A five-year study

**DOI:** 10.3892/ol.2014.2501

**Published:** 2014-09-04

**Authors:** MERVAT HESHAM, MERVAT ATFY, TAMER HASSAN, MOHAMED ABDO, SAED MORSY, MOHAMED EL MALKY, DALIA ABDEL LATIF

**Affiliations:** Pediatrics Department, Zagazig University, Zagazig 44111, Egypt

**Keywords:** solid tumors, lymphoma, epidemiology, children

## Abstract

Worldwide, the incidence and mortality rates of childhood cancers differ. The study of incidence patterns and survival rates in childhood malignancies is important in aiding in the planning of treatment centers and in obtaining further information with regard to the etiology. Few studies have investigated the survival in cases of childhood solid tumors in Egypt. The aim of the current study was to evaluate the patterns, frequency and outcome of solid tumors and lymphomas in children admitted to and followed up at the Pediatric Oncology Department of Zagazig University Hospital (Zagazig, Egypt) over a duration of 5 years (January 2004 to December 2008). A retrospective study was conducted, which included 155 children with solid tumors and lymphomas. The medical records were reviewed and the relevant data collected, in particular, those concerning demographic, clinical, histopathological, laboratory and imaging data as well as the treatment plans and outcomes. The mean age of patients was 5.6±3.04 years at diagnosis. The patients comprised 94 males and 61 females. Non-Hodgkin lymphoma (NHL) was the most common tumor type, followed by neuroblastoma (31.0 and 29.0%, respectively). When patients were stratified in terms of age (<5, ≥5 but <10, and ≥10 years), the <5-years-of-age group exhibited the greatest number of patients. Fever, pallor and pain were the most frequent initial clinical presentations among the patients and stage II was the most common stage (39.1%) followed by stage IV, III and I (35.0, 20.3 and 5.6% respectively). The overall 5-year survival rate in the study group was 66.7%. The survival rate was significantly higher in patients with Wilm’s tumor and Hodgkin lymphoma, followed by NHL (92.0, 88.0 and 72.0%, respectively; P<0.001), while the mortality rate was significantly higher in patients with neuroblastoma (P<0.001). In conclusion, NHL and neuroblastoma were the most common tumors; the survival rates were higher in patients with Wilm’s tumor and Hodgkin lymphoma and lower in patients with neuroblastoma. A larger multicenter study is required to further investigate the conclusions drawn from this study.

## Introduction

Cancer is the leading cause of mortality in children between the ages of 6 months and 15 years in the USA and annually >7,000 new cases of cancer are diagnosed in children <15 years of age (1).

Leukemia comprises ~25% of all childhood cancers, followed by tumors of the central nervous system (17%), neuroblastoma (7%), non-Hodgkin lymphoma (NHL) (6%), Wilm’s tumor (6%), Hodgkin disease (5%), rhabdomyosarcoma (3%), retinoblastoma (3%), osteosarcoma (3%) and Ewing sarcoma (2%). Numerous additional rare tumor types account for the remainder. These values are with regard to patients ≤15 years of age, worldwide (2).

During the past 30 years, improvements in the survival rates for infants and children with different types of cancer have been observed. In the 1960s, ~50% of all children with malignant tumors succumbed to the disease, compared with a 75% overall survival rate today (3). The improved outcomes were associated with the establishment of well-designed national multidisciplinary cooperative studies, which were conducted in various pediatric cancer centers, using combined treatment modalities, including surgery, chemotherapy and irradiation (4). The availability of new imaging techniques was also significant in identifying the extent of tumors and determining staging. The most improved relative survival rates have been achieved in patients with acute lymphocytic leukemia, NHL and Wilm’s tumor (3).

The aim of the current study was to evaluate the patterns, frequency and outcome of solid tumors and lymphomas in children admitted to and followed up in the Pediatric Oncology Department of Zagazig University Hospital (Zagazig, Egypt) over a duration of 5 years (January 2004–December 2008).

## Materials and methods

A retrospective study was conducted, which included 155 children with solid tumors and lymphomas who were admitted to and followed up in this department between January 2004 and December 2008.

All medical records were reviewed and a standardized data abstraction form was designed to capture the appropriate information with regard to the demographic, clinical, histopathological, laboratory and imaging data, as well as the treatment plan and outcome.

### Statistical analysis

The data obtained for each patient were added to a computerized database in SPSS, version 14 (SPSS, Inc., Chicago, IL, USA). A descriptive analysis was performed for each variable included and correlations between these variables, and the outcome were analyzed using the appropriate statistical method. Data are presented as the mean ± standard deviation for quantitative variables, and as a value and percentage for qualitative variables. The χ^2^, analysis of variance (f) tests were used where appropriate, and P<0.05 was considered to indicate a statistically significant difference.

### Ethics

The study was performed in accordance with the ethical standards of the 1964 Declaration of Helsinki, as revised in 2000 (5). The study was approved by the ethics committee of Zagazig University (Zagazig, Eygpt).

## Results

The frequency of tumors, age groups and gender distributions are shown in Table I. The mean age of patients at diagnosis was 5.6±3.04 years. The most commonly involved age group was <5 years (60.0%) and the least common was >10 years (12.3%). Neuroblastoma was the most common cancer in early childhood (38.7% of tumors in children <5 years old), while NHL occurred most frequently in the older age groups (39.5% of tumors in children aged between 5 and 10 years old and 36.8% of tumors in children ≥10 years old).

**Figure 1:**
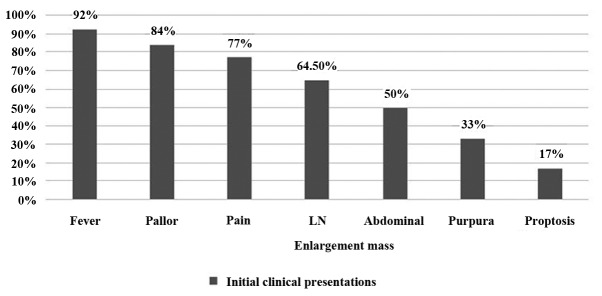
Initial clinical presentations of patients: Fever, pallor and pain were the most common. LN, lymph node.

The patients comprised 94 males (60.6%) and 61 females, (39.4%), with a male to female ratio of 1.5:1. NHL was the most common tumor in males (35.1%), whilst for females, it was neuroblastoma (37.7%). For the entire cohort, NHL was the most prevalent followed by neuroblastoma (31.0 and 29.0%, respectively). The most frequently observed primary tumor sites were the abdomen (41.3%), head and neck (22.6%) and mediastinum (16.7%). Fever, pallor and pain were the most common initial clinical presentations among the patients (Fig. 1; 91.6, 83.9 and 77.4%, respectively).

Of the 155 patients, the tumor stage was only known for 143 cases. Stage II was the most common stage (39.1%) followed by stage IV, III and I (35.0, 20.3 and 5.6% respectively) (Table II). Complete results with regard to the outcome were available for 129 patients. The overall 5-year survival rate was 66.7%. No significant differences were identified between the various age groups and between males and females with regard to the 5-year survival rate (P>0.05). Conversely, a significant correlation was observed between tumor stage and the 5-year survival rate, where the 5-year survival rate was significantly higher in patients with stages I and II (100 and 84%, respectively; P<0.001) and significantly lower in stage IV patients (43%; P<0.001) (Table III). The correlation between the 5-year survival rate and tumor type is shown in Table IV. The 5-year survival rate was significantly higher in patients with Wilm’s tumor and Hodgkin lymphoma followed by NHL (92, 88 and 72%, respectively; P<0.001), while the mortality rate was significantly higher in patients with neuroblastoma (P<0.001).

## Discussion

Current statistics on the occurrence and outcome of cancer cases are essential for the planning and evaluation of programs for cancer control. The present study collected a database of pediatric malignant solid tumors and lymphomas from the only reference pediatric center in the Zagazig region of Egypt, with the aim of analyzing the patterns, frequency and outcomes of these tumors.

The study investigated 155 children with malignant solid tumors and lymphomas over a duration of 5 years, yielding a mean of 31 cases per year. An exact incidence rate cannot be provided by a study based in a single hospital (Pediatrics Department, Zagazig University); however, the information is useful in revealing patterns of childhood malignancies in this region.

In the current study, NHL was the most common tumor followed by neuroblastoma and Hodgkin lymphoma (31.0, 29.0 and 17.4%, respectively). Agboola *et al* (6) reported 77 children with malignant tumors, and observed that lymphomas were the most prevalent malignancy identified, accounting for 31 diagnoses (40%). Burkitt’s lymphoma accounted for the majority of malignancies (28 cases; 36%), followed by retinoblastoma (16 cases; 21%) and Wilm’s tumor (11 cases; 14%).

Following the exclusion of leukemia, a report from a hospital-based registry by the Italian Association of Pediatric Hematology and Oncology demonstrated that NHL was the most common tumor (7). Memon *et al* (8) investigated 113 cases of malignant solid tumors in children; the results indicated that retinoblastoma was the most common malignant solid tumor, followed by Wilm’s tumor (38.9% and 13.2%, respectively). Furthermore, Akinde *et al* (9) in their retrospective review on diagnosed cases of childhood tumors observed between January 2000 and 2007 from the records of Lagos University Teaching Hospital in Nigeria, found that malignant tumors constituted 30.50% of childhood tumors of which retinoblastoma was the most common.

The low incidence of brain tumors in the current study was due to the referral of the majority of patients with brain tumors to the Neurosurgery Department, where surgery and radiotherapy were the recommended treatment options. This was also observed in patients with bone tumors.

The current study revealed male preponderance, with an overall male to female ratio of 1.5:1. This is marginally lower than that reported in another Egyptian study (10) where the male to female ratio was 1.6:1, and slightly higher than that reported in a Turkish study (11), where the male to female ratio was 1.4:1. However, the ratio observed in the current study was significantly higher than the 1.1:1 reported in a Mexican study (12).

The majority of the patients included in the current study were <5 years of age at diagnosis (58.7%). Juárez-Ocaña *et al* (12)found that the highest frequency of cancer was identified in the group of 1- to 4-year-olds (36.8%). Furthermore, the results presented in the current study, with regards to the age of patients at diagnosis, are consistent with a number of other studies (7,13,14).

The majority of patients in the current study initially presented with fever, pallor and pain (91.6, 83.9, and 77.4%, respectively). Lymph node enlargement was the most frequent symptom among children with lymphoma and abdominal mass was the most common among children with neuroblastoma and Wilm’s tumor. Gao *et al* (15) reported that the initial symptoms among 63 patients with advanced neuroblastoma included fever, abdominal pain, abdominal mass and leg or articular pain.

The majority of patients in the current study were stage II (39.1%), followed by stage IV, III and I (35, 20.3 and 5.6%, respectively). The frequency of cancer stages had been previously analyzed in a number of studies (12,16,17). In a large Mexican study (12), a total of 1,702 new cases of cancer in children were registered between 1996 and 2001. The majority of cases were stage III (36.8%) followed by stage IV, II and I (30.1, 19.6 and 13.5%, respectively). A high percentage (66.9%) of Mexican children were of stage III or IV at the time of diagnosis, which is marginally higher than the 55.7% of patients with stage III or IV cancer in the present study. The increased incidence of advanced-stage cases in the present study and that by Juárez-Ocaña *et al* (12) was explained by the unawareness of general practitioners and parents of the early symptoms of these types of cancer.

The overall 5-year survival rate in the current study was 66.7%. Vasudevan *et al* (14) reported a 10-year overall survival rate of 41.5%. According to the National Cancer Institute (NCI) (16), the 5-year survival rate for all childhood cancers (including leukemia) combined is nearing 81%. Inclusion of leukemia in NCI results may explain the increased 5-year survival rate in NCI compared with the current study.

The 5-year survival rate was significantly higher in patients with Wilm’s tumor and Hodgkin lymphoma, followed by NHL (92.0, 88.0 and 72.0%, respectively), while the mortality rate was significantly higher in patients with neuroblastoma. These results are consistent with a number of studies (14,16,17). Since the 1990s, the 5-year survival rates for Wilm’s tumor and Hodgkin lymphoma have exceeded 90% (17). The 5-year survival rate for children <15 years of age with NHL in the USA has improved significantly, increasing from 45% between 1975 and 1978, to 88% between 1999 and 2002, which is slightly higher than that observed in the current study (17). Furthermore, Smith *et al* (17) reported that the 5-year survival rates have improved for older children (>1 year) with neuroblastoma in the USA from ~40% prior to 1985, to 65% between 1999 and 2002, which is relatively low compared with other childhood cancers. The high mortality rate in neuroblastoma in the current study may be attributed to the late stage of the disease at diagnosis, as stages III and IV represented 63.3% of neuroblastoma cases.

In conclusion, NHL and neuroblastoma were the most common tumors. The survival rate was higher in patients with Wilm’s tumor and Hodgkin lymphoma, and lower in patients with neuroblastoma. A larger multicenter study is required to confirm these results. The low incidence of brain and bone tumors in the present study is due to the lack of communication with neurosurgeons and orthopedic surgeons who are involved primarily in the treatment of these tumors and hence establishment of tumor boards must include neurosurgeons and orthopedic surgeons to ensure accurate registration and team management of patients with brain and bone tumors.

## Figures and Tables

**Table I:** Demographic characteristics of patients stratified by tumor type.

			Age groups, n (%)			Gender, n (%)	
				
Tumor	Frequency, n (%)	Mean age, y	<5 y	≥5, <10 y	≥10 y	Male	Female
NHL	48 (31.0)	6.6±4.1	24 (25.8)	17 (39.5)	7 (36.8)	33 (35.1)	15 (24.6)
Neuroblastoma	45 (29.0)	3.7±2.1	36 (38.7)	7 (16.3)	2 (10.5)	22 (23.4)	23 (37.7)
HL	27 (17.4)	6.2±3.9	15 (16.1)	10 (23.3)	2 (10.5)	17 (18.1)	10 (16.4)
Wilm’s tumor	15 (9.7)	4.9±2.3	8 (8.6)	7 (16.3)	0 (0.0)	8 (8.5)	7 (11.7)
Rhabdomyosarcoma	8 (5.2)	6.8±3.9	4 (4.3)	1 (2.3)	3 (15.8)	5 (5.3)	3 (4.8)
Bone tumors	7 (4.5)	10±4.5	2 (2.2)	0 (0.0)	5 (26.3)	5 (5.3)	2 (3.2)
Brain tumors	3 (1.9)	3.3±2.3	2 (2.2)	1 (2.3)	0 (0.0)	2 (2.2)	1 (1.6)
Hepatoblastoma	2 (1.3)	1.2±0.4	2 (2.2)	0 (0.0)	0 (0.0)	2 (2.2)	0 (0.0)
Total number	155	-	93	43	19	94	61
Test	-	F=5.24		χ^2^=25.12		χ^2^=6.02	
P-value	-	P<0.001		P<0.05		P>0.05	

NHL, Non-Hodgkin lymphoma; HL, Hodgkin lymphoma; y, year. P<0.001 vs. different tumor types; P<0.05 vs. different tumor types; P>0.05 vs. different tumor types.

**Table II:** Tumor stages in the patients.

Tumor	Stage I, n (%)	Stage II, n (%)	Stage III, n (%)	Stage IV, n (%)
NHL	1 (12.5)	21 (37.5)	14 (48.3)	12 (24.0)
HL	4 (50.0)	11 (19.6)	6 (20.7)	6 (12.0)
Neuroblastoma	0 (0.0)	15 (26.8)	6 (20.7)	20 (40.0)
Wilm’s tumor	1 (12.5)	6 (10.7)	1 (3.4)	4 (8.0)
Rhabdomyosarcoma	2 (25.0)	0 (0.0)	0 (0.0)	6 (12.0)
Brain tumors	0 (0.0)	1 (1.8)	1 (3.4)	0 (0.0)
Bone tumors	0 (0.0)	2 (3.6)	1 (3.4)	1 (2.0)
Hepatoblastoma	0 (0.0)	0 (0.0)	0 (0.0)	1 (2.0)
Total number	8	56	29	50

NHL, Non-Hodgkin lymphoma; HL, Hodgkin lymphoma.

**Table III:** Correlation between the outcome and various parameters.

	Age group, n (%)			Gender, n (%)		Stage, n (%)			
			
Outcome	<5 y	≥5, <10 y	≥10 y	Male	Female	Stage I	Stage II	Stage III	Stage IV
Survivors	49 (62.0)	26 (76.0)	11 (69.0)	55 (70.0)	31 (62.0)	8 (100.0)	43 (84.0)	16 (61.5)	19 (43.0)
STD	30 (38.0)	8 (24.0)	5 (31.0)	24 (30.0)	19 (38.0)	0	8 (16.0)	10 (38.5)	25 (57.0)
Total number	79	34	16	79	50	8	51	26	44
Test		χ^2^=2.27			χ^2^=0.8			χ^2^=22.37	
P value		P>0.05			P>0.05			<0.001	

y, years; STD, succumbed to the disease. P>0.05 vs. distribution of outcome variables.

**Table IV:** Correlation between the outcome and various types of tumors.

Outcome	NHL	Neuroblastoma	HL	Wilm’s	Rhabdomyosarcoma	Brain tumor	Bone tumor	Hepatoblastoma
Survivors	31 (72)	12 (34)	23 (88)	11 (92)	4 (67)	1 (100)	3 (75)	1 (50)
STD	12 (28)	23 (66)	3 (12)	1 (8)	2 (33)	0	1 (25)	1 (50)
Total number	43	35	26	12	6	1	4	2
Test					χ^2^=26.89			
P-value					P<0.001			

Data are presented as n (%). NHL, non-Hodgkin lymphoma; HL, Hodgkin lymphoma; STD, succumbed to the disease. P<0.01 vs. different tumor types.
